# Genome-Based Microsatellite Development in the *Culex pipiens* Complex and Comparative Microsatellite Frequency with *Aedes aegypti* and *Anopheles gambiae*

**DOI:** 10.1371/journal.pone.0013062

**Published:** 2010-09-30

**Authors:** Paul V. Hickner, Becky deBruyn, Diane D. Lovin, Akio Mori, Susanta K. Behura, Robert Pinger, David W. Severson

**Affiliations:** 1 Eck Institute for Global Health, Department of Biological Sciences, University of Notre Dame, Notre Dame, Indiana, United States of America; 2 Department of Physiology and Health Science, Ball State University, Muncie, Indiana; American Museum of Natural History, United States of America

## Abstract

**Background:**

Mosquitoes in the *Culex pipiens* complex are among the most medically important vectors for human disease worldwide and include major vectors for lymphatic filariasis and West Nile virus transmission. However, detailed genetic studies in the complex are limited by the number of genetic markers available. Here, we describe methods for the rapid and efficient identification and development of single locus, highly polymorphic microsatellite markers for *Cx. pipiens* complex mosquitoes via *in silico* screening of the *Cx. quinquefasciatus* genome sequence.

**Methodology/Principal Findings:**

Six lab colonies representing four *Cx. pipiens* and two *Cx. quinquefasciatus* populations were utilized for preliminary assessment of 38 putative loci identified within 16 *Cx. quinquefasciatus* supercontig assemblies (CpipJ1) containing previously mapped genetic marker sequences. We identified and validated 12 new microsatellite markers distributed across all three linkage groups that amplify consistently among strains representing the complex. We also developed four groups of 3–5 microsatellite loci each for multiplex-ready PCR. Field collections from three cities in Indiana were used to assess the multiplex groups for their application to natural populations. All were highly polymorphic (Mean  = 13.0 alleles) per locus and reflected high polymorphism information content (PIC) (Mean  = 0.701). Pairwise F_ST_ indicated population structuring between Terre Haute and Fort Wayne and between Terre Haute and Indianapolis, but not between Fort Wayne and Indianapolis. In addition, we performed whole genome comparisons of microsatellite motifs and abundance between *Cx. quinquefasciatus* and the primary vectors for dengue virus and malaria parasites, *Aedes aegypti* and *Anopheles gambiae*, respectively.

**Conclusions/Significance:**

We demonstrate a systematic approach for isolation and validation of microsatellites for the *Cx. pipiens* complex by direct screen of the *Cx. quinquefasciatus* genome supercontig assemblies. The genome density of microsatellites is greater in *Cx. quinquefasciatus* (0.26%) than in *Ae. aegypti* (0.14%), but considerably lower than in *An. gambiae* (0.77%).

## Introduction

Mosquitoes in the *Culex pipiens* complex are major vectors for a number of important human pathogens including West Nile virus, St. Louis encephalitis virus, and *Wuchereria bancrofti*, a causative agent of lymphatic filariasis [1]–[3]. *Cx. pipiens* complex mosquitoes can be found on every continent except Antarctica [4], and include two widespread species: *Cx. quinquefasciatus* (Say 1823) and *Cx. pipiens* (Linnaeus 1758). *Cx. quinquefasciatus* inhabits tropical, subtropical, and warm temperate zones while *Cx. pipiens* inhabits temperate zones [4]. Sympatric populations occur where their ranges overlap [5], [6]. A recent study on the *Cx. pipiens* complex along a north-south transect in North America revealed hybrid populations as far north as Illinois and as far south as Alabama [6]. Distinct physiological differences between species in the *Cx. pipiens* complex are known and are thought to influence pathogen transmission as well as their geographic distribution [7], [8]. Identification of the genes contributing to these physiological processes could provide novel targets for genetic control methods. Though genetic marker development has facilitated construction of detailed linkage maps in dengue virus vector, *Aedes aegypti* [9]–[12] and the *Plasmodium falciparum* vector, *Anopheles gambiae* [13]–[16] mosquitoes, genetic studies based on linkage analyses in *Cx. pipiens* are limited due to the paucity of available marker loci [8], [17].

Microsatellites have been preferred as genetic markers due to their high polymorphism, co-dominance, and potential for high throughput analysis. Approximately 33 microsatellite markers have been developed for *Cx. pipiens* complex to date [18]–[21], yet none of these have been mapped to their respective linkage group. One disadvantage of microsatellite markers is they typically need to be developed for each species of interest [22]. Indeed, cross-species utility of individual microsatellite loci within the *Cx. pipiens* complex can be limited [20]. Because spatial distributions of individual *Cx. pipiens* complex members often overlap across freely interbreeding hybrid zones [5], [6], population studies would benefit greatly with availability of additional microsatellites loci with broad species compatibility.

Here we employ a method we recently reported for *Ae. aegypti* [23] toward the rapid and efficient development of microsatellite markers for *Cx. pipiens* complex mosquitoes by screening *Cx. quinquefasciatus* whole genome shotgun sequence (wgs) supercontig assemblies for microsatellite motifs [24]. We utilize 12 microsatellites to assess structuring among *Cx. pipiens* populations from three cities in Indiana, USA. Additionally, we perform a comparative genome analysis of microsatellite repeat motifs and abundance in *Cx. pipiens*, *An. gambiae* and *Ae. aegypti*.

## Materials and Methods

### Ethics Statement

Our protocol for maintenance and care of experimental animals was reviewed and approved by the Institutional Animal Care and Use Committee at the University of Notre Dame. Animals are maintained and cared for in the Freimann Life Science Center, an AAALAC accredited facility.

### *In silico* identification of microsatellites

Sequences previously mapped using restriction fragment length polymorphism (RFLP) markers based on random cDNAs [8], [17] were used for BLASTn analysis against the *Cx. quinquefasciatus* whole genome sequence assembly (CpipJ1) at VectorBase [24]. Genome supercontigs containing the RFLP marker sequences were downloaded from VectorBase and screened with Tandem Repeats Finder (TRF) software using the default parameters [25]. TRF output data were evaluated for regions containing microsatellites with a period size of 2–4 bp and number of repeats less than 30.

### Primer design

Sequences of 400–600 bp containing a microsatellite of interest were extracted from individual supercontigs and subjected to BLASTn analysis against the *Cx. quinquefasciatus* genome at VectorBase to determine the degree of repetitive sequences flanking the target microsatellite sequence. PCR primers were designed for those regions with minimal repetitive sequences using Primer3 v.4.0 [26], with a target amplicon size of 120–400 bp. The resulting primer sequences were subjected to BLASTn analysis against the *Cx. quinquefasciatus* genome to assess copy number and potential nontarget amplification. Primer adjustments were made with the assistance of OligoCalc [27]. In addition, BLASTn analysis was performed using PCR primer and amplicon sequences of previously described microsatellite loci [18]–[21] against the *Cx. quinquefasciatus* genome at VectorBase to determine if any of these loci were within supercontigs with known genetic map positions based on RFLP marker loci [8], [17].

### Mosquito samples

Individuals from six laboratory colonies were utilized in the preliminary screening of all microsatellite loci. These included four colonies of *Cx. pipiens* (Gose, Shinkura, Shasta and South Bend strains) and two of *Cx. quinquefasciatus* (Boana and Johannesburg strains). Shinkura is an autogenous strain founded from samples collected in 1998 from Tokushima, Japan. The South Bend and Gose strains are described elsewhere [8]. The Johannesburg strain was the source for the *Cx. quinquefasciatus* genome project [24]. The Shasta and Boana strains were kindly provided by Anton Cornel, University of California at Davis.

**Figure 1:**
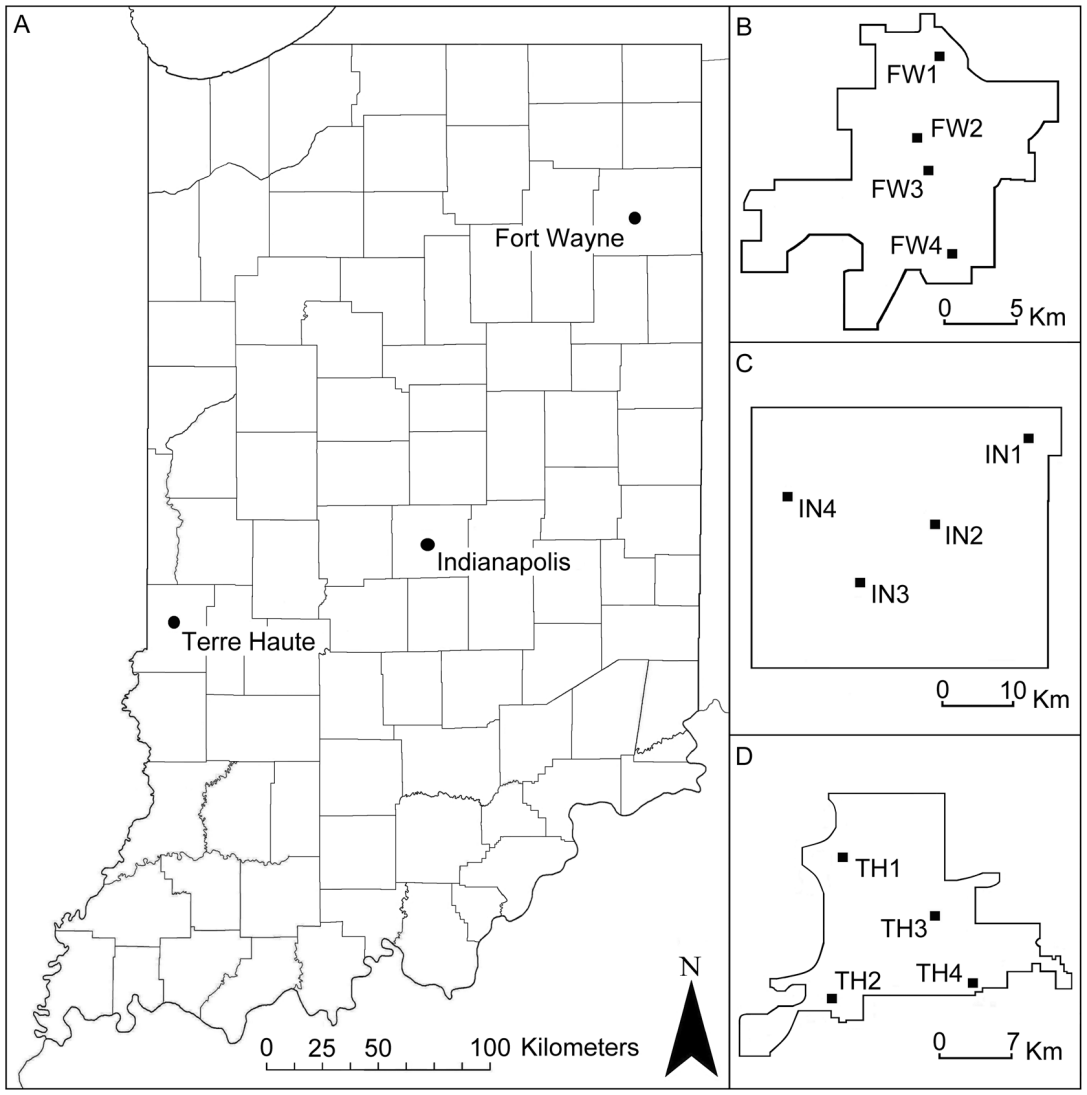
Maps showing the spatial relationships among the three cities sampled in Indiana and locations of the collection sites within city boundaries. A: City locations in Indiana. B: Fort Wayne. C: Indianapolis. D: Terre Haute. Coordinates listed in Table S1.

Field samples of *Cx. pipiens* populations were collected from three cities in Indiana (Fort Wayne, Indianapolis, and Terre Haute) during August-October, 2008 (Figure 1). Four collection sites within each city were chosen according to recommendations from local health department personnel (Figure 1, Table S1). Distances between cities ranged from ∼260 km (Ft. Wayne to Terre Haute) to ∼110 km (Indianapolis to Terre Haute). Egg rafts were collected using oviposition traps made from 17 L plastic pans (Sterilite) containing an aged (3–5 days) alfalfa-infusion [28]. Larvae (∼20 from each egg raft) were reared in 8 oz. plastic deli cups with ∼150 mL of aged tap water and fed a slurry of water and crushed Tetramin® fish food (Spectrum Brands Co.). Larvae were reared to the 3^rd^ or 4^th^ instar at which time they were identified to species level [29] and preserved in 95% ethanol.

### DNA extraction and PCR amplification

DNA extractions on individuals from the laboratory colonies were performed using a simple alkaline method [30]. DNA from each alkaline extraction was suspended in a final volume of 1500 µL containing 0.01 M NaOH and 0.018 M Tris-HCL, pH 8.0. Genomic DNA extractions from field samples were performed using the DNeasy® Tissue Kit (Qiagen) or a standard phenol/chloroform method [31]. Only one larva from an egg raft was extracted for PCR amplification.

PCR amplification was performed in 25 µL reactions in 96-well PCR plates (Dot Scientific). Each reaction contained 1X Taq buffer (50 mM KCl, 10 mM Tris pH 9.0, 0.1% Triton X), 1.5 mM MgCl_2_, 200 µM dNTPs, 5 pmoles each primer, 1 unit of Taq DNA polymerase, and 1 µL of genomic DNA (∼20 ng). Thermal cycling was performed using Mastercycler® thermocyclers (Eppendorf) under the following conditions: initial denaturation for 5 min at 94°C followed by 30 cycles of denaturation for 1 min at 94°C, annealing for 1 min at 60°C, extension for 2 min at 72°C, with a final extension for 10 min at 72°C.

### Preliminary screening and multiplex PCR

Preliminary assessment of microsatellites for PCR amplification and copy number was performed by size fractionation of PCR products by electrophoresis in 2% agarose gels stained with ethidium bromide and visualized using UV light. Microsatellites amplifying across at least five of the six lab strains with single copy amplicons were assessed for allelic polymorphism on 6% denaturing polyacrylamide gels using the GenePrint® STR System (Promega). Sequences of single-copy microsatellite loci were submitted to the GenBank STS database (Table S2). Multiplexes were assembled according to amplicon size and tested on two individuals from each lab strain (n = 12) and three individuals from each of the three cities sampled (n = 9) by size fractionation using the 3730 Genetic Analyzer (Applied Biosystems) [32]. Mendelian inheritance was assessed based on conformity to Hardy-Weinberg expectations in samples from the Johannesburg (n = 28) and South Bend (n = 28) colonies.

### Genotyping

Fluorophore-labeled (6-FAM®, HEX®, NED®) forward primers were used in PCR amplification for fragment analysis as described above. Prior to fragment analysis, multiplex PCR products were diluted 1∶10 in sterile H_2_O, and 1 µL of this dilution was added to 9 µL of a mixture containing 1 mL HiDi Formamide® (Applied Biosystems) and 15 µL ROX 400HD® (Applied Biosystems) in 96-well PCR plates. The samples were denatured for 2 minutes at 94°C and immediately placed on ice. Amplification products were sized using the ABI PRISM 3730 Genetic Analyzer (Applied Biosystems) and ROX 400HD size standard. Alleles were called using GENEMAPPER® v.4.0 software (Applied Biosystems), with subsequent visual verification of each sample.

### Comparative genome analysis

The whole genome sequence assemblies of *Cx. quinquefasciatus* (CpipJ1.2), *Ae. aegypti* (AaegL1.1) and *An. gambiae* (AgamP3.4) were scanned for microsatellite motifs using the SciRoKo3.4 software program [33]. Our search was restricted to identify perfect di-, tri-, tetra-, penta- and hexa-nucleotide repeats with no less than 7, 5, 4, 4 and 4 repeats in each, respectively. The flanking regions of microsatellites (200 bp either side) were extracted using the ‘Little Helper’ module of SciRoKo. The absolute counts and average motif lengths in each microsatellite category obtained from the program output were used to calculate the relative proportions of microsatellite sequences in each genome.

### Statistical analysis

Genetic diversity among the field populations was based on the observed and expected heterozygote frequencies and the number of alleles at each locus. ARLEQUIN v3.0 [34] was used to calculate F_IS_, pairwise F_ST_ and AMOVA following Weir and Cockerham [35] and to perform an exact test of Hardy-Weinberg (HW) equilibrium following Guo and Thompson [36]. GENEPOP 4.0 [37], [38] was used to test for isolation by distance using a Mantel test. Polymorphism information content (PIC) was calculated using Excel Microsatellite Toolkit [39]. The BOTTLENECK software program was used to assess the microsatellite data for evidence of recent population reductions based on gene diversity and allele frequency distributions [40].

## Results

### Development of microsatellite loci

From our preliminary screening of 16 *Cx. quinquefasciatus* whole genome sequence supercontigs containing previously mapped RFLP marker loci [8], [17] we selected 38 putative microsatellite loci for further evaluation. These represented five dinucleotide, eight trinucleotide and 1 tetranucleotide motifs (Table 1). The majority of microsatellites were dinucleotide repeats, and among these, the TG/AC motif was the most common. We identified 12 loci within eleven supercontigs that amplified consistently, were single copy and polymorphic when tested in individuals from six laboratory colonies representing both *Cx. pipiens* and *Cx. quinquefasciatus* populations derived from diverse sites worldwide (Table S3). Of these 12 loci, 9 (75%) comprise dinucleotide repeats, while the remaining three loci comprise trinucleotide repeats. Another 21 microsatellites, while single copy, showed strain-specific amplification, were monomorphic or did not have alleles within HW expectations thus limiting their potential utility across the species complex. With the remaining five microsatellites, no amplification was obtained with any of the strains. Only one of the 16 supercontigs examined (3.626) did not contain microsatellite motifs. BLASTn analysis of microsatellites against *Cx. quinquefasciatus* transcripts (CpipJ1.2 Gene Build) indicated that C99TGT1 and C177GAA1 were within CPIJ005634 and CPIJ008257, respectfully, while no other microsatellites were within coding regions [24]. Additionally, BLASTn analysis indicated that none of the previously reported microsatellites were within these 16 supercontigs, but that two of them are located in other supercontigs with known genetic marker loci (CxpGT4, gb-AY423738, supercontig 3.5, chromosome 2-36.1; CxqTri4, gb-AY958079, supercontig 3.208, chromosome 3-26.3) [24]. To improve the throughput and minimize the cost of microsatellite genotyping, individuals from the six laboratory colonies and *Cx. pipiens* field collections were used to develop four PCR multiplexes consisting of 3–5 microsatellite primer sets (Table 2). These include one locus on chromosome 1, seven loci on chromosome 2, and four loci on chromosome 3. Except for C48GTT1 and C48CGA1, which are on the same supercontig, the loci on chromosome 2 are distributed across 76.4 cM out of a total of 85.9 cM, while the the loci on chromosome 3 are distributed across 47.9 cM out of a total of 79.2 cM (see Table S3). The number of repeats, number of alleles and heterozygosities observed per locus are similar to that observed for *Cx. pipiens* microsatellites described previously [7], [18]–[21], [41].

**Table 1:** Microsatellite loci PCR screen results categorized by repeat motif.

			Strain-specific	No
	Polymorphic	Monomorphic	Amplification	Amplification
Repeat	(n = 15)	(n = 5)	(n = 11)	(n = 6)
Dinucleotide repeats				
AG/TC	2		1	
AT/TA			1	1
CA/GT	3		2	1
CT/GA	1		1	1
TG/AC	6	2	3	3
Trinucleotide repeats				
ATC/TAG			1	
CAA/GTT	1			
CGA/GCT			1	
CGC/GCG	1			
CGT/GCA		1		
GAA/CTT		1		
GAC/CTG	1			
TGT/ACA			1	
Tetranucleotide repeats				
ACAT/TGTA		1		

**Table 2:** Multiplex-ready PCR groups.

Group	Microsatellite locus	Map location^a^	Predicted amplicon size (bp)^b^	Fluorochrome
CX1	C177CA1	2-76.4	130	HEX®
	C68GA1	2-9.6	154	56-FAM®
	C127TC1	1-0.0	178	NED®
	C99TGT1	3-17.9	214	HEX
	C65AC1	2-15.9	305	56-FAM
CX2	C205TG1	3-18.5	150	56-FAM
	C134AC1	2-42.3	195	NED
	C48GTT1	2-29.2	328	HEX
CX3	C48CGA1	2-29.2	137	HEX
	C68GA1	2-9.6	154	56-FAM
	C32AC1	2-00.0	184	NED
CX4	C139TG1	3-26.0	201	56-FAM
	C127TC1	1-0.0	178	NED
	C446AC2	3-65.8	256	HEX

a Genetic map position after Mori et al. [8], [17].

b Size based on *Cx. quinquefasciatus* genome sequence [24].

**Figure 2:**
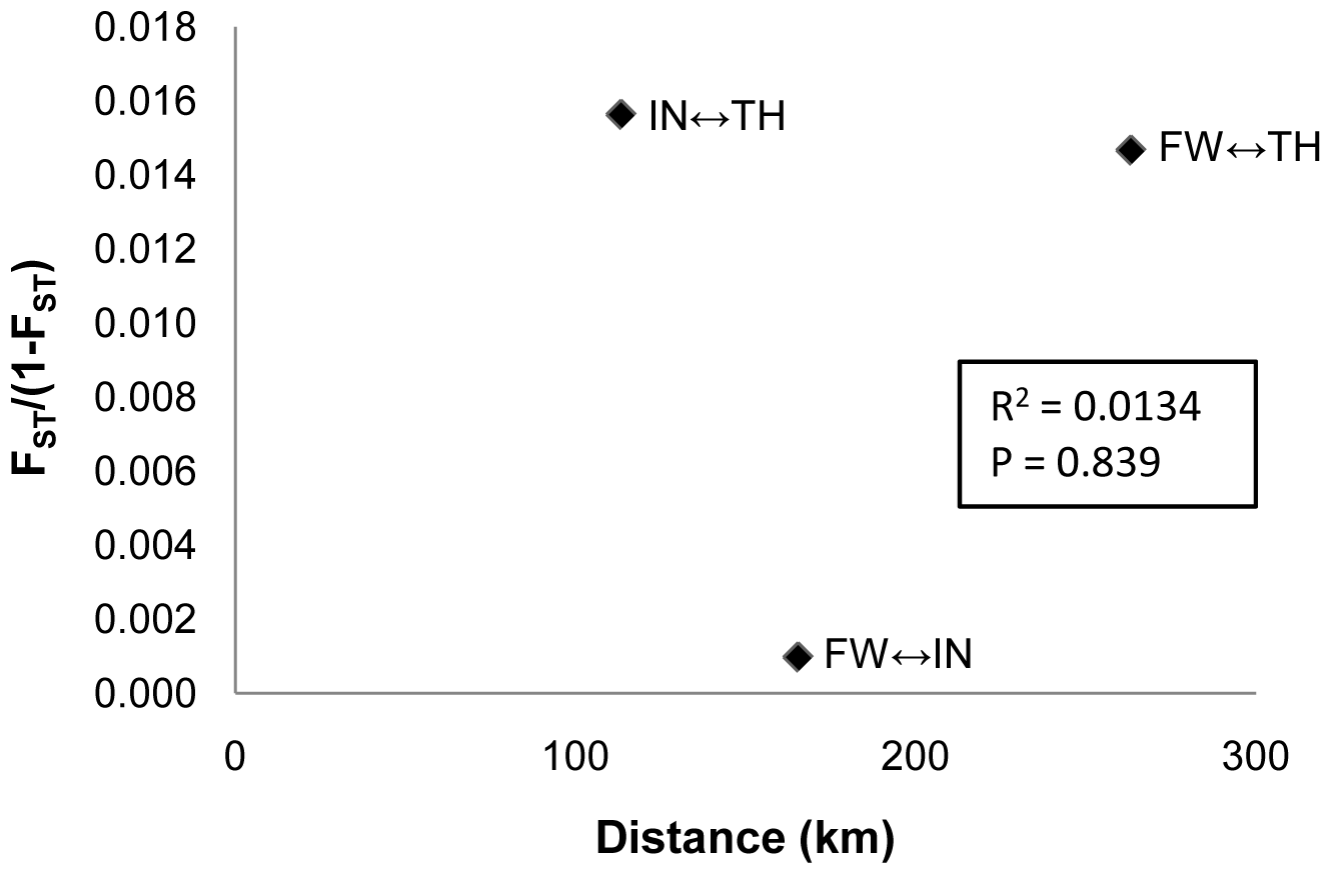
Regression analysis of pairwise FST/(1-FST) against pairwise linear distances between three cities sampled in Indiana, USA.

A panel of 12 microsatellites was used to assess the population structure among *Cx. pipiens* populations from three cities in Indiana (n = 266 individuals). All of the loci were highly polymorphic with 7 to 25 alleles (mean  = 13.0) and PIC ranging from 0.509 to 0.889 (mean  = 0.701) (Table 3). Allele frequencies of eight loci (C177CA1, C68GA1, C127TC1, C99TGT1, C65AC1, C48GTT1, C48CGA1, and C205TG1) were within HW expectations in all three cities, while four loci (C139TG1, C446AC2, C32AC1, and C134AC1) were within HW expectations in at least one city (Table 4). Generally, F_IS_ was lower in Fort Wayne and Indianapolis populations than in Terre Haute (Table S4). Though pairwise F_ST_ values were relatively low among the three cities, significant structuring was evident between populations from Fort Wayne and Terre Haute and between populations from Indianapolis and Terre Haute, yet virtually no structuring was evident between populations from Fort Wayne and Indianapolis (Table 5). Tests for recent population bottlenecks based on gene diversities and allele frequency distributions were not significant. Results of AMOVA indicated that 97.02%, 1.88% and 1.10% of the estimated genetic variation was within individuals of a population, within populations, and among populations, respectively. No significant correlation between F_ST_/(1-F_ST_) and distance (Figure 2) was detected suggesting there was no isolation by distance (R^2^ = 0.0134, p = 0.839).

**Table 3:** Characterization of 12 microsatellite markers from *Cx. pipiens* collections (n = 266) from 3 cities in Indiana, USA.

Locus	Repeat Motif	No. of Alleles	Allele Size Range	PIC^a^
C177CA1	(CA)_12_	18	111–152	0.760
C68GA1	(GA)_8_	14	122–155	0.794
C127TC1	(CT)_39_	11	96–129	0.787
C99TGT1	(TGT)_6_	10	184–226	0.759
C65AC1	(AC)_13_	13	266–299	0.684
C205TG1	(TG)_12_	14	114–170	0.697
C134AC1	(AC)_7_	7	185–199	0.554
C48GTT1	(GTT)_6_	9	307–337	0.766
C48CGA1	(CGA)_9_	7	117–135	0.570
C32AC1	(AC)_11_	13	207–222	0.798
C139TG1	(TG)_10_	25	198–241	0.889
C446AC2	(AC)_7_	15	217–255	0.579

a PIC: allelic polymorphism information content.

**Table 4:** Summary statistics for microsatellite markers from each city representing study sites in Indiana.

	Fort Wayne (n = 86)		Indianapolis (n = 93)		Terre Haute (n = 87)	
Locus	H_O_	H_E_	H_O_	H_E_	H_O_	H_E_
C177CA1	0.732	0.761	0.739	0.792	0.693	0.799
C127TC1	0.812	0.802	0.753	0.818	0.750	0.817
C99TGT1	0.798	0.801	0.806	0.806	0.742	0.759
C65AC1	0.639	0.709	0.736	0.735	0.747	0.706
C205TG1	0.674	0.634	0.677	0.673	0.609	0.611
C139TG1	0.779	0.864	0.785*	0.881	0.609*	0.861
C134AC1	0.639	0.529	0.538*	0.463	0.793*	0.656
C48GTT1	0.578	0.631	0.772	0.750	0.682	0.725
C48CGA1	0.588	0.578	0.615	0.607	0.591	0.620
C446AC2	0.553	0.573	0.618	0.640	0.477*	0.590
C32AC1	0.733	0.773	0.802	0.818	0.682*	0.800
C68GA1	0.779	0.800	0.742*	0.825	0.881	0.822

H_O_  =  observed heterozygote frequency,

H_E_  =  expected heterozygote frequency under HW expectations,

* =  significant deviation from HW expectations (p≤0.05).

**Table 5:** Pairwise F_ST_ estimates among three cities in Indiana, USA.

	Fort Wayne	Indianapolis
**Indianapolis**	0.0010	
**Terre Haute**	0.0155*	0.0166*

*Significant after permutation test [34].

Comparative genome analysis indicated that the relative abundance of microsatellite sequences varies among the three mosquitoes (Table 6). Our results show that smaller repeat motifs (di, tri-or tetra-nucleotide repeats) are more frequent in the *Cx. quinquefasciatus* genome (0.153%) than in the *Ae. aegypti* genome (0.109%), but considerably less frequent in both these genomes compared to the *An. gambiae* (0.75%) genome. The microsatellites with larger motifs (the penta- and hexa-nucleotide repeats) are relatively more frequent in the *Cx. quinquefasciatus* genome (0.109%) compared to that in the *Ae. aegypti* (0.035%) or the *An. gambiae* (0.022%) genome. However, the overall density of microsatellite sequences is relatively lower in the *Cx. quinquefasciatus* (2.6 kb/Mb) or the *Ae. aegypti* genomes (1.4 kb/Mb) compared to that in the *An. gambiae* genome (7.7 kb per Mb).

**Table 6:** Genome-wide microsatellite representation in *Cx. quinquefasciatus*, *Ae.aegypti*, and *An. gambiae*.

	Total Number			Mean # Repeats			Total Content (%)		
Motif categories	*Culex*	*Aedes*	*Anopheles*	*Culex*	*Aedes*	*Anopheles*	*Culex*	*Aedes*	*Anopheles*
Dinucleotide	15689	7657	47857	24.49	37.80	25.38	0.066	0.022	0.437
Trinucleotide	19615	35705	31639	17.95	20.03	22.05	0.061	0.055	0.251
Tetranucleotide	7119	18207	7970	21.10	22.67	21.68	0.026	0.032	0.062
Pentanucleotide	4063	8716	1093	56.11	30.55	27.34	0.039	0.020	0.011
Hexanucleotide	4673	3941	423	86.81	50.88	72.62	0.070	0.015	0.011
**Total**	51159	74226	88982	41.29	32.44	33.81	0.263	0.144	0.771

## Discussion

Prior to our study there were ∼33 microsatellites available for *Cx. pipiens* complex mosquitoes [18]–[21]. However, not all of those amplify in both *Cx. pipiens* and *Cx. quinquefasciatus*, and even fewer amplify in *Cx. pipiens pallens* [20]. Furthermore, information on chromosome location is not currently available for those loci. Here we identified and validated 12 new microsatellites with broad application to *Cx. pipiens* complex mosquitoes, bringing the total number of microsatellites available to ∼45. All amplified in the six laboratory strains tested (Table S3), which includes colonies representing *Cx. quinquefasciatus*, *Cx. pipiens* (anautogenous), *Cx. pipiens* (autogenous) and *Cx. pipiens* from Japan (often referred to as *Cx. pipiens pallens*). Because we identified and scanned supercontigs containing markers previously mapped to chromosome locations, the relative locations of our microsatellites are known. An *a priori* assumption often made in studies of genetic variation is that the marker loci used provide reasonable coverage across the genome. Also, few problems were encountered while developing the PCR multiplexes with up to five primers sets, thus indicating the potential for the development of multiplexes other than those reported here. As expected, most primer sets worked well when genotyping our Johannesburg colony, but adjustments to primer sequences were often necessary to obtain consistent amplification (i.e., expected allele frequencies) in our South Bend strain. Once primers were adjusted for consistent amplification in both the South Bend and the Johannesburg colonies, they would usually work well for genotyping the field collections. However, several microsatellites worked well in our laboratory colonies but still reflected high null allele frequencies when they were tested on the field collections (Table S3). Nonetheless, these markers still have potential for use genetic mapping in laboratory colonies or investigating *Cx. pipiens* complex populations from other geographic areas.

Population structure among our study sites was consistent with findings from a similar study in which Huang et al. characterized 11 *Cx. pipiens* populations in the northeast United States using 12 microsatellites and detected significant structuring among several urban and rural populations with F_ST_ ranging from 0.0101 to 0.0174 in anautogenous *Cx. pipiens* [41]. However, Kothera et al. genotyped *Cx. pipiens* and *Cx. quinquefasciatus* populations from 14 sites in a north-south transect and reported significant F_ST_ ranging from 0.003 to 0.318 [6]. The observed structuring among populations from the three cities sampled in this study (F_ST_ ranging from 0.001 to 0.016) was due to significant differences between the Terre Haute population and both the Fort Wayne and Indianapolis populations. Because the local health departments in Indianapolis and Terre Haute were applying insecticide to potential *Culex* breeding sites during the period in which we collected our samples, we used the software program BOTTLENECK to determine if we could detect any recent population bottlenecks based on our microsatellite data. However, tests for recent reductions in the effective population sizes were not significant.

This study represents the first attempt to characterize microsatellite sequence representation in the *Cx. quinquefasciatus* assembled genome. Moreover, our comparative analysis provides a better understanding of genome-wide abundance of microsatellites among *Cx. quinquefasciatus, Ae. aegypti* and *An. gambiae*. It is well-known that microsatellite frequency is low in *Ae. aegypti* compared to several other insects, including *An. gambiae*. We note that an earlier study by Meglécz et al. [42] examined whole genome sequence (WGS) traces, but not the assembled genomes. Our results were consistent with these results after accounting for the respective differences in search strategies. That is, they also included mono-nucleotide repeats in their analysis and they used lower thresholds for di- and tri- nucleotide repeats than our thresholds for such repeats. Moreover, our estimate of microsatellites in *An. gambiae* closely match results of another study [43]. Microsatellites seem to be considerably more abundant (in terms of percentage of sequences per genome) in *An. gambiae* than in either *Ae. aegypti* or *Cx. quinquefasciatus*. On the other hand, the *Cx. quinquefasciatus* genome has a higher frequency of microsatellites with larger motifs (the penta- and hexa-nucleotide repeats) indicating a possible expansion of microsatellite motif length in this mosquito.

In conclusion, here we demonstrate a systematic approach for the isolation and validation of microsatellites for *Cx. pipiens* complex mosquitoes by screening *Cx. quinquefasciatus* genome supercontig assemblies for short tandem repeats. We tested and validated 12 microsatellites that amplified consistently and were polymorphic in lab colonies of *Cx. quinquefasciatus* and *Cx. pipiens* representing six populations from three continents. Additionally, microsatellite allele frequencies were within HW expectations in samples from the Johannesburg and South Bend colonies. We used four PCR multiplexes to assess the population structure of *Cx. pipiens* from three cities in Indiana, USA, thus demonstrating their usefulness for studies on natural populations. Lastly, we performed comparative genome analysis to characterize microsatellite type and abundance in three major disease vectors, *An. gambiae*, *Ae. aegypti*, and *Cx. pipiens* mosquitoes. The development of microsatellites using this approach could provide additional genetic markers to produce a linkage map with moderate resolution for *Cx. pipiens* mosquitoes and provide a foundation for further genetic analyses such as QTL mapping and population structure analysis. Moreover, this demonstrates the potential for the development of microsatellites from existing genome sequences for other closely related taxa.

## Supporting Information

Table S1 Coordinates for collection sites in Fort Wayne (FW), Indianapolis (IN), and Terre Haute (TH), Indiana, USA.
(0.04 MB DOC)

Table S2 GenBank accession numbers for STS sequences of microsatellite loci.
(0.06 MB DOC)

Table S3 Microsatellite variation among lab strains.
(0.11 MB DOC)

Table S4 F_IS_ estimates for each locus in each city sampled.
(0.04 MB DOC)
