# Supplementary material for: Genome-Based Microsatellite Development in the Culex pipiens Complex and Comparative Microsatellite Frequency with Aedes aegypti and Anopheles gambiae
Source: PLoS One. 2010 Sep 30;5(9):e13062. doi: 10.1371/journal.pone.0013062 (PMC2948009; doi:10.1371/journal.pone.0013062)
Supplement: Table S1 — Coordinates for collection sites in Fort Wayne (FW), Indianapolis (IN), and Terre Haute (TH), Indiana, USA. (0.04 MB DOC) [file pone.0013062.s001.doc]

**Table S1.** Coordinates for collection sites in Fort Wayne (FW), Indianapolis (IN), and Terre Haute (TH), Indiana, USA.

|  |  |  |
| --- | --- | --- |
| **Collection site** | **Latitude** | **Longitude** |
| FW1 | N41°09'32.84" | W85°07'36.18" |
| FW2 | N41°06'13.00" | W85°09'09.22" |
| FW3 | N41°05'11.09" | W85°08'29.72" |
| FW4 | N41°01'17.00" | W85°07'34.75" |
|  |  |  |
| IN1 | N39°53'31.89" | W86°58'59.56" |
| IN2 | N39°47'44.66" | W86°05'55.67" |
| IN3 | N39°43'54.51" | W86°11'20.55" |
| IN4 | N39°49'25.55" | W86°17'04.69" |
|  |  |  |
| TH1 | N39°29'48.80" | W87°24'44.46" |
| TH2 | N39°25'31.32" | W87°25'47.67" |
| TH3 | N39°27'53.64" | W87°20'28.39" |
| TH4 | N39°25'56.39" | W87°19'52.16" |
