# Supplementary material for: Genome-Based Microsatellite Development in the Culex pipiens Complex and Comparative Microsatellite Frequency with Aedes aegypti and Anopheles gambiae
Source: PLoS One. 2010 Sep 30;5(9):e13062. doi: 10.1371/journal.pone.0013062 (PMC2948009; doi:10.1371/journal.pone.0013062)
Supplement: Table S2 — GenBank accession numbers for STS sequences of microsatellite loci. (0.06 MB DOC) [file pone.0013062.s002.doc]

**Table S2.** GenBank accession numbers for STS sequences of microsatellite loci.

| **SSR locus** | **GenBank accession #** |
| --- | --- |
| C127GAC1 | GF102017 |
| C32AC1 | GF102018 |
| C32TC1 | GF102019 |
| C68GA1 | GF102020 |
| C65TG1 | GF102021 |
| C65AC1 | GF102022 |
| C65CGC1 | GF102023 |
| C474CT1 | GF102024 |
| C48GTT1 | GF102025 |
| C66CA1 | GF102026 |
| C177CA1 | GF102027 |
| C177TG1 | GF102028 |
| C205CA1 | GF102029 |
| C205TG1 | GF102030 |
| C139TG1 | GF102031 |
| C68ACAT1 | GF102032 |
| C68CA1 | GF102033 |
| C48ATC1 | GF102034 |
| C48CGA1 | GF102035 |
| C175AT1 | GF102036 |
| C134AC1 | GF102037 |
| C129GT1 | GF102038 |
| C66GA1 | GF102039 |
| C99TC1 | GF102040 |
| C99TGT1 | GF102041 |
| C446AC2 | GF102042 |
| C446TG1 | GF102043 |
| C32TG1 | GF102044 |
| C175TG1 | GF102045 |
| C127TC1 | GF110607 |
| C139CGT1 | GF110608 |
| C177GAA1 | GF110609 |
| C127GA1 | GF110610 |
|  |  |

.
