# Supplementary material for: Genome-Based Microsatellite Development in the Culex pipiens Complex and Comparative Microsatellite Frequency with Aedes aegypti and Anopheles gambiae
Source: PLoS One. 2010 Sep 30;5(9):e13062. doi: 10.1371/journal.pone.0013062 (PMC2948009; doi:10.1371/journal.pone.0013062)
Supplement: Table S3 — Microsatellite variation among lab strains. (0.11 MB DOC) [file pone.0013062.s003.doc]

**Table S3.** Microsatellite variation among lab strains.

| **Microsatellite locus^a^** | | **Supercontig #** | **Genetic locus^b^** | **GenBank accession #** | **Map location^c^** | | **Predicted amplicon size (bp)** | | **Forward primer 5'-3'** | | | **Reverse primer 5'-3’** | | |  | | |
| --- | --- | --- | --- | --- | --- | --- | --- | --- | --- | --- | --- | --- | --- | --- | --- | --- | --- |
| **Single copy, polymorphic and validated in lab strains and field populations (n = 12)** | | | | | | | | | | | | | | |  | | |
| C32AC1 [11] | | 3.32 | LF334 | BM005506 | 2-00.0 | | | 184 | CGATGCCTTCCGCAAGATC | | GAGTGGATTGTTAGAATGCGC | | | |  | | |
| C48CGA1 [9] | | 3.48 | CX40 | FD664709 | 2-29.2 | | | 137 | CGATGTGATTTGTACCCTTCGC | | GGCGGCCGTTTCTGTTGTC | | | |  | | |
| C48GTT1 [6] | | 3.48 | CX40 | FD664709 | 2-29.2 | | | 328 | GTGGGTCACTTCGAAGGACACC | | CTGGGAACCGTTGGCTGATCC | | | |  | | |
| C65AC1 [13] | | 3.65 | CX90 | FD664719 | 2-15.9 | | | 305 | GGAGTTGTGCGGTTGAAAGT | | GCACTGCCTAACGGATCATT | | | |  | | |
| C68GA1 [8] | | 3.68 | LF335 | BM005505 | 2-9.6 | | | 154 | ACACGTGGTGCGATGATCC | | ATCAGCTGATGGTAACCCAGA | | | |  | | |
| C99TGT1 [6] | | 3.99 | CX112 | FD664727 | 3-17.9 | | | 214 | GCTGTCATCGCCGAAGAAGT | | GCAACCACAACCACAAGTCG | | | |  | | |
| C127TC1 [39] | | 3.127 | CX60 | FD664718 | 1-0.0 | | | 178 | GCTGACTGGAAGTAGCGACAA | | GCAATCAAGCGTGGCCATTAA | | | |  | | |
| C134AC1 [7] | | 3.134 | CX61 | FD664712 | 2-42.3 | | | 195 | TGAAGGTCAGCCACTCAGGC | | ACAGCTGACTCTCGTCGACA | | | |  | | |
| C139TG1 [10] | | 3.139 | CX53 | FD664714 | 3-26.0 | | | 201 | GAGCTGTCATTCTTGGAGGC | | CGTCCATTTTTCCACGTTCGAC | | | |  | | |
| C177CA1 [12] | | 3.177 | CX114 | FD664728 | 2-76.4 | | | 130 | CGTTTGCTTCTCGCACCTCA | | CAGCACAAACATCATCAGGGA | | | |  | | |
| C205TG1 [12] | | 3.205 | CX17 | FD664699 | 3-18.5 | | | 150 | CGGTTGACTTTTCGTCGCTGT | | CATGGGCCACGGTCATATCC | | | |  | | |
| C446AC2 [7] | | 3.446 | CX11 | FD664697 | 3-65.8 | | | 256 | GTGAAGTGGGTGAAGATTAGC | | CGCGTTTATTCCGGCTTCG | | | |  | | |
| **Single copy and polymorphic with allele frequencies within HW expectations in two lab colonies (n = 3)** | | | | | | | | | | | | | | |  | | |
| C65CGC1 [8] | | 3.65 | CX90 | FD664719 | 2-15.9 | | | 221 | TCTGGGTACAACCCCGTAAC | | AGAGAGTGCGCAAAAGCAAT | | | |  | | |
| C127GA1 [10] | | 3.127 | CX60 | FD664718 | 1-0.0 | | | 159 | CGATTCGGAACGGAACGAC | | ACCTAACTCGTCTGCAAAGCC | | | |  | | |
| C474CT1 [6] | | 3.474 | DDC | U27581 | 2-27.3 | | | 147 | CCCAAACTTGCCACAAAAGT | | CACCCACCACACCGTTTATGC | | | |  | | |
| **Single copy and polymorphic in lab strains (n = 6)** | | | | | | | | | | | | | | |  | | |
| C127GAC1 [8] | | 3.127 | CX60 | FD664718 | 1-0.0 | | | 307 | GCGTTTGGAGAGTGGAAAAG | | TGAGTTTTCAGTGCCCTCCT | | | |  | | |
| C32TC1 [14] | | 3.32 | LF334 | BM005506 | 2-00.0 | | | 198 | CGCATGCAATCAAAGGAGGC | | ATCCAGCGAGTATGTGAGCAC | | | |  | | |
| C65TG1 [9] | | 3.65 | CX90 | FD664719 | 2-15.9 | | | 302 | ACTGCGAAACGCTTACTGCT | | GTGTGTGGACTGTGGTGGAG | | | |  | | |
| C66CA1 [6] | | 3.66 | CX35 | FD664707 | 2-65.9 | | | 213 | CGACTACTGCCCCAATTTGT | | CACCCTCCCCTACAGACGTA | | | |  | | |
| C177TG1 [8] | | 3.177 | CX114 | FD664728 | 2-76.4 | | | 197 | AGCACAAAAAGGCACGATTT | | TAAACGCAAGTAGGCGGAGT | | |  | |  | |
| C205CA1 [9] | | 3.205 | CX17 | FD664699 | 3-18.5 | | | 227 | CAATGCGCCTTCTGGATTAT | | CTCGTGATGGCCATTTCTCT | | |  | | | |
| **Single copy monomorphic or strain-specific amplification (n=12)** | | | | | |  | | |  |  | | | |  | | |  |
| C32TG1 [11] | | 3.32 | LF334 | BM005506 | 2-00.0 | | 400 | | CGTGTTTTCCATTGTTGGTG | | TTGGCTGTGTCAACTGCTTC | | B, J, SA^d^ | | | |  |
| C68ACAT1 [6] | | 3.68 | LF335 | BM005505 | 2-9.6 | | 425 | | GGCCTTGCTGAGAAAACTTG | | CCCAAAATCCAAGCTTCAAA | | B, J, SA | | | |  |
| C68CA1 [7] | | 3.68 | LF335 | BM005505 | 2-9.6 | | 294 | | ATAAAGCGACCAAGGCTCAA | | GCGAAACCATTCAAAAGCAT | | B, J, SA, SI, SB | | | |  |
| C48ATC1 [10] | | 3.48 | CX40 | FD664709 | 2-29.2 | | 337 | | CATTTTTCGGGTGGCTTCTA | | CGAGATCGAAATGATGCTGA | | B, G, J, SA, SI | | | |  |
| C175AT1 [13] | | 3.175 | LF129 | BM005504 | 2-41.2 | | 185 | | GGACCAAGGGTACGATTTGA | | CAGACTGGTTAACGGCTTCC | | B, G, J, SA, SB | | | |  |
| C175TG1 [14] | | 3.175 | LF129 | BM005504 | 2-41.2 | | 295 | | TCAGATCTCCGAGAGGAGGA | | CTGTCAGGGCCAGATTTCAT | | J, SA, SB | | | | |
| C129GT1 [15] | | 3.129 | CX107 | FD664723 | 2-54.3 | | 377 | | AAGGTGCAAAACCAAACTGG | | TGGAGCACAGCCCTACTCTT | | B, SA, SI, SB | | | | |
| C66GA1 [12] | | 3.66 | CX35 | FD664707 | 2-65.9 | | 333 | | ATGCTCACGAGTGAGGGTTC | | ATAAGAGCCCAAACGCAGAA | | J | | | | |
| C99TC1 [19] | | 3.99 | CX112 | FD664727 | 3-17.9 | | 233 | | GCGGGCAGAGTCAAAAAGTA | | GCGAAAAAGGACAAGAACGA | | B, G, J, SA, SB | | | | |
| C446TG1 [7] | | 3.446 | CX11 | FD664697 | 3-65.8 | | 397 | | GGAAAGGGGCACTTGTGTAA | | CGTTTGCTTCTCTTCGAACC | | B, G, J, SA | | | | |
| C139CGT1[7] | | 3.139 | CX53 | FD664714 | 3-26.0 | | 207 | | ACATGGGACTCTCGATGGTGA | | CGAGTTGGTCTTCTGCTGGAC | | Amplified in all 6 | | | | |
| C177GAA1[7] | | 3.177 | CX114 | FD664728 | 2-76.4 | | 161 | | CCGAGCGACCCAATCCAAC | | CGTAATGTCGCTGTCTTCGTAC | | Amplified in all 6 | | | | |
| **No amplification or no usable microsatellites identified (n=5)** | | | | | | | | |  |  | | |  | | | |  |
| C474AC1 [7] | 3.474 | | DDC | U27581 | 2-27.3 | | 205 | | AAACGCTTTTCCCTTCTTCC | | GTTGTTCAACACCCCTCCAT | |  | | | |  |
| C134TG1 [5] | 3.134 | | CX61 | FD664712 | 2-42.3 | | 251 | | CGAAAAGCGAGAGTTGGTTC | | CCATCTCAAAGTCCCTCGAA | |  | | | |  |
| C129TA1 [19] | 3.129 | | CX107 | FD664723 | 2-54.3 | | 384 | | ATCGCGAGGTGAAAAGAGAA | | GAAAAACAACCCTCGAATGAA | |  | | | |  |
| C66GT1 [6] | 3.66 | | CX35 | FD664707 | 2-65.9 | | 256 | | TCTTTTTCGCTGTGGCTTTT | | ACATAACCTCGTGGGCAAAG | |  | | | |  |
| none | 3.626 | | CX22 | FD664703 | 2-65.8 | |  | |  | |  | |  | | | |  |
| ^a^[ ]: number of repeats; ^b^Genetic locus associated with supercontig; ^c^Genetic map position after Mori et al. [8,17]; ^d^Strain-specific amplification, B=Boana, G=Gose, | | | | | | | | | | | | | | | | |  |
| J=Johannesburg, SA=Shasta, SI=Shinkura, SB=South Bend. | | | | | | | | | | | | | | | | |  |
