# Supplementary material for: Genome-Based Microsatellite Development in the Culex pipiens Complex and Comparative Microsatellite Frequency with Aedes aegypti and Anopheles gambiae
Source: PLoS One. 2010 Sep 30;5(9):e13062. doi: 10.1371/journal.pone.0013062 (PMC2948009; doi:10.1371/journal.pone.0013062)
Supplement: Table S4 — FIS estimates for each locus in each city sampled. (0.04 MB DOC) [file pone.0013062.s004.doc]

**Table S4.** F_IS_ estimates for each locus in each city sampled.

| **Locus** | **Fort Wayne** | **Indianapolis** | **Terre Haute** |
| --- | --- | --- | --- |
| C177CA1 | 0.038 | 0.068 | 0.068 |
| C127TC1 | -0.013 | 0.081 | 0.081 |
| C99TGT1 | 0.004 | -0.001 | -0.001 |
| C65AC1 | 0.100 | -0.002 | -0.002 |
| C205TG1 | -0.064 | -0.006 | -0.006 |
| C139TG1 | 0.099 | 0.110 | 0.110 |
| C134AC1 | -0.209 | -0.162 | -0.162 |
| C48GTT1 | 0.084 | -0.029 | -0.029 |
| C48CGA1 | -0.018 | -0.015 | -0.015 |
| C446AC2 | 0.035 | 0.034 | 0.034 |
| C32AC1 | 0.052 | 0.019 | 0.019 |
| C68GA1 | 0.027 | 0.101 | 0.101 |
| **All** | 0.020 | 0.024 | 0.058 |
|  |  |  |  |
